# Psychoanalytic Perspectives on the Psychological Effects of Stillbirth on Parents: A Protocol for Systematic Review and Qualitative Synthesis

**DOI:** 10.3389/fpsyg.2020.01216

**Published:** 2020-06-19

**Authors:** Loredana Cena, Alberto Stefana

**Affiliations:** Department of Clinical and Experimental Sciences, University of Brescia, Brescia, Italy

**Keywords:** stillbirth, psychoanalisis, psychoanalic therapy, clinical cases, protocol for systematic review, parents

## Abstract

**Introduction:** Despite the fact that stillbirth has a broad economic impact on health systems and society and despite the fact that the importance of appropriate psychological and social support for parents has been highlighted, there is still a lack of research exploring the intrapsychic and interpersonal dynamics and issues triggered by the experience of stillbirth. Healthcare professionals attempting to provide effective psychological support to bereaved parents who have suffered perinatal loss continue to struggle to achieve better and deeper understanding of their psychological states and processes. Psychoanalysis could play a key role in improving this situation, but the studies available are confined to journals of psychoanalysis, and there is a lack of synthesis, leaving this knowledge beyond the reach of scientists from other theoretical approaches or disciplines. This protocol proposes the systematic review and qualitative synthesis of articles from journals of psychoanalysis on the psychological effects on parents of stillbirth.

**Methods and Analysis:** This systematic review will follow, as far as possible, the Preferred Reporting Items for Systematic Reviews and Meta-Analyses (PRISMA) guidelines. The Psychoanalytic Electronic Publishing Archive (1999–2019), the Single Case Archive (1999–2019), and PsycINFO (1999–2019) will be used to identify relevant articles. The review will include articles reporting clinical material and/or theoretical considerations concerning parent psychological states and processes triggered by the experience of stillbirth, and a meta-synthesis will be performed.

**Ethics and Dissemination:** Formal ethical approval is not required for this study, as no primary data will be collected. The findings will be published in an international peer-reviewed journal.

## Strengths and Limitations of This Study

• This will be the first systematic review of the psychoanalytic literature on the psychological effects of stillbirth on parents.
• Psychoanalysis represents the most coherent and intellectually satisfying model to explicate human behavior.
• This study protocol follows the recommendations by the PRISMA protocols.
• Given the characteristics of both single-case psychoanalysis reports and theoretical considerations, quality assessment is not applicable.
• The data analyzed and synthesized will include descriptions, explanations, and interpretations made by the authors of published articles.

## Introduction

### Background

The World Health Organization (2014) defines stillbirth as “the birth of a baby with no signs of life at or after 28 completed weeks of pregnancy.” According to this definition, more than 2.5 million of stillborn occurred globally each year (Blencowe et al., 2016). Furthermore, when taking into account deaths occurring in the second trimester of pregnancy, such estimate increases by 32% (Smith et al., 2018).

Stillbirth is a devastating life event (Koopmans et al., 2013; Burden et al., 2016) that has a profound, negative, and long-lasting impact on parents socially, economically, and psychologically (Heazell et al., 2016; Murphy and Cacciatore, 2017). This traumatic experience often leads to complicated reactions of grief that can negatively affect parents’ mental and physical health and well-being (Kersting and Wagner, 2012). Furthermore, although the normal grieving process usually lasts about 2 years, the majority of women conceive within 1 year of the loss (Hughes et al., 1999), and the subsequent pregnancy is often conditioned by the negative psychological effects of the stillbirth, even with the birth of a healthy child (Blackmore et al., 2011). Additionally, it has been extensively reported that there is an increased incidence of relationship breakdown and parent separation or divorce following a stillbirth (Turton et al., 2009; Gold et al., 2010; Shreffler et al., 2012).

### Gaps in the Empirical Literature

Despite the fact that stillbirth has a broad economic impact on health systems and society (Heazell et al., 2016) and despite the fact that the importance of appropriate psychological and social support for parents has been highlighted—see, for example, the two comprehensive international Stillbirth Series launched by *The Lancet* in 2011 and 2016, which identified stillbirth as one of the “most shamefully neglected” (Darmstadt, 2011) areas of public health and called for a “global consensus on a package of care after a death in pregnancy or childbirth … for the affected family, community and caregiver” (de Bernis et al., 2016)—there is still a lack of research exploring the intrapsychic and interpersonal dynamics and issues triggered by the experience of stillbirth. To date, the empirical literature has mainly focused on predictors of mental health and well-being for women only (Homer et al., 2016), and household surveys have been the main source of data (Temmerman and Lawn, 2018). Very few studies have explored the experience in depth from the parents’ point of view, with the partial exception of certain qualitative studies that investigate the mother’s experiences (Ellis et al., 2016). As a consequence, those attempting to provide effective psychological support in perinatal bereavement, the success of which is vital for preventing negative short- and long-term outcomes for families (Heazell et al., 2016), continue to struggle to achieve better and deeper understanding of the psychological processes (emotions, needs, beliefs, desires) of bereaved parents who have suffered a perinatal loss. Such a deeper level of understanding could be used as a source of information for training, guidelines, and bereavement care principles.

### The Psychoanalytic Approach to the Study of the Stillbirth

Psychoanalytic literature has made original and important contributions to describing the psychological dynamics of people who have experienced traumatic events (e.g., Khan, 1963; Baranger et al., 1988; Laub and Auerhahn, 1993; Hesse and Main, 1999; Bohleber, 2007; Gamba and Stefana, 2016; Stefana and Gamba, 2016; Stefana and Lavelli, 2016; Stefana et al., 2017). Nevertheless, contributions by psychoanalysis have been excluded from the mainstream of the scientific community devoted to the study of effects of stillbirth on parents’ mental health. However, if we consider the current weak areas in the empirical research of the psychological impact of stillbirth on families, it is clear that further investigation is needed into the psychological dynamics of parents of stillborn children. A psychoanalytic approach to this issue—since psychoanalysis represents the most coherent and intellectually satisfying model to explicate human behavior (Kandel, 1999, 2012; Fonagy and Allison, 2016; Spiro, 2020), particularly the influence of unconscious on psychological functioning (Kernberg and Michels, 2016)—could be key to finding solutions, but the studies available are not broadly disseminated in scientific literature (as they are usually confined to journals of psychoanalysis), and there is a lack of synthesis, putting this knowledge beyond the reach of scientists from other theoretical approaches or disciplines.

With regard to psychoanalysis, it is important to remember that single-case reports are quintessential in terms of theory, research, and practice (Desmet et al., 2013; Stefana, 2015, 2017). Single-case reports are definable as the therapists’ narrative report of what happened during one or more sessions or the entire therapeutic journey through interpretations based on clinical experience with their own patients (Iwakabe and Gazzola, 2009). The case study method is a valuable method based on clinical experience rather than on replicable scientific evidence, with close examination of the details of a patient’s personal/clinical history (Gomm et al., 2000; Hinshelwood, 2013); it is the method of choice for studying a phenomenon in context (Willemsen et al., 2017), especially when there is the wish to explore and describe the deepest levels of the human psyche (Dodes and Dodes, 2017), which could include those affected by a stillbirth.

## Objective

The objective of this systematic review is to synthesize psychoanalytic knowledge (derived from both clinical practice and theoretical research) regarding mothers’ and fathers’ intrapsychic and interpersonal dynamics and issues triggered by the experience of stillbirth.

## Methods and Analysis

The Preferred Reporting Items for Systematic Reviews and Meta-Analyses (PRISMA)-P guidelines (Shamseer et al., 2015) were used to prepare this protocol. As far as possible, this review will follow the PRISMA guidelines (Moher et al., 2009).

### Protocol Registration

This systematic review protocol was submitted for registration to the International Prospective Register of Systematic Reviews (PROSPERO) on January 6, 2020 (ID number 163198).

### Study Searches

Searches will be performed by two reviewers using the Psychoanalytic Electronic Publishing Archive (PEP-Web Archive), Single Case Archive (SCA), and PsycINFO [only for those journals included in the category “Psychology, Psychoanalysis” of ISI Journal Citation Report (JCR)]. The following search term combinations will be used: “stillbirth” OR “stillbirths” OR “still-birth” OR “still-births” OR “stillborn” OR “stillborns” OR “still-born” OR “still-borns” OR “intrauterine death” OR “intrapartum death” OR “fetal death.” A full-text search will be performed in the PEP-Web Archive, while the search will be limited to Title/Topic in the SCA (with a further limitation to Psychodynamic/Psychoanalytic Theoretical Orientation) and PsycINFO. A manual search will also be performed in the references of selected articles. These searches will be limited to English-language articles published from January 1, 1999, to December 31, 2019.

The PEP-Web Archive contains the full texts of 74 premier journals in psychoanalysis, with over 119,000 articles dated from 1871 to 2019; the SCA contains around 500 single-case psychoanalysis studies published in ISI-ranked journals; and the ISI JCR contains 13 journals, three of which are not included in the PEP-Web Archive.

### Inclusion Criteria

Every article presenting clinical material (derived from individual, couple, family, or group psychoanalysis or psychoanalytic psychotherapy) and/or theoretical considerations on the psychological implications of stillbirth for parents will be included in this review.

### Quality Assessment

Given the characteristics of both single-case psychoanalysis reports (Hinshelwood, 2013) and theoretical considerations, quality assessment is not applicable.

### Screening

The full text of the search results will be independently screened by two reviewers to identify articles that meet the inclusion criteria. Any disagreement over the eligibility of a particular article will be resolved through discussion until a consensus is reached.

### Data Abstraction

The article will be read by one reviewer who will delineate discrete thematic units, i.e., passages in the original text where a clinical and/or theoretical description of the psychological effects of the stillbirth experience is presented. All the thematic units will then be collated into a single Microsoft Word document.

### Data Analysis

We will adopt a sequential approach to synthesizing the thematic units from this review. However, in line with the Cochrane Qualitative and Implementation Methods Group (CQIMG) guidance and advice (Booth et al., 2016), since we cannot establish the best method of data synthesis until we know the breadth and depth of the data reported in the included studies, we will select an appropriate method of synthesis after the data abstraction.

### Patient and Public Involvement

No patients will be involved in this systematic review.

### Ethics and Dissemination

Formal ethical approval is not required for this study, as no primary data will be collected. The findings of this review will be published in an international peer-reviewed journal.

## Author Contributions

No person other than the two authors listed here has contributed to the manuscript preparation. LC was the project’s principal investigator and agreed to be responsible for all aspects of the study. AS contributed to study conception and design, and drafted the study report and revised it. Both authors contributed to the article and approved the submitted version.

## Conflict of Interest

The authors declare that the research was conducted in the absence of any commercial or financial relationships that could be construed as a potential conflict of interest.
